# Low expression of SPARC in gastric cancer-associated fibroblasts leads to stemness transformation and 5-fluorouracil resistance in gastric cancer

**DOI:** 10.1186/s12935-019-0844-8

**Published:** 2019-05-21

**Authors:** Yongchen Ma, Jing Zhu, Shanwen Chen, Ju Ma, Xiaoqian Zhang, Sixia Huang, Jianwen Hu, Taohua Yue, Junling Zhang, Pengyuan Wang, Xin Wang, Long Rong, Hongjie Guo, Guowei Chen, Yucun Liu

**Affiliations:** 1 Department of General Surgery, Peking University First Hospital, Beijing, 100034 People’s Republic of China; 2 Department of Pathology, Peking University First Hospital, Beijing, 100034 People’s Republic of China; 3 Department of Endoscopic Center, Peking University First Hospital, Beijing, 100034 People’s Republic of China; 4 Department of Interventional Radiology and Vascular Surgery, Peking University First Hospital, Beijing, 100034 People’s Republic of China

**Keywords:** SPARC, Cancer-associated fibroblasts, Gastric cancer, Stemness, Drug resistance

## Abstract

**Background:**

The aim of the present study was to clarify the correlations between SPARC expression in gastric cancer-associated fibroblasts (GCAFs) and the prognosis of patients with gastric cancer and to elucidate the role of GCAF-derived SPARC in stemness transformation and 5-fluorouracil resistance in gastric cancer.

**Methods:**

One hundred ninety-two patients were enrolled in the present study. SPARC expression levels were evaluated by immunohistochemical staining. Primary GCAFs were obtained and cultured from cancer patients for in vitro study, and a lentivirus infection method was employed to knock down SPARC expression in GCAFs. The stemness phenotype and 5-fluorouracil (5-FU) response of gastric cancer cells were assessed via a 3D co-culture model. The apoptotic status and stemness alterations were monitored by flow cytometry and western blotting. Additionally, label-free quantification proteomics was used to identify the differentially expressed proteins and potential pathways in gastric cancer cells treated with GCAF-derived SPARC.

**Results:**

Low expression of GCAF-derived SPARC was associated with decreased differentiation and reduced 5-year overall survival and was an independent predictive factor for prognosis in gastric cancer. The 3D tumour growth and 5-FU resistance abilities of gastric cancer cells were elevated after treatment with GCAFs with SPARC knockdown relative to these abilities in negative control cells. Additionally, suppressing SPARC expression in GCAFs facilitated the phenotypic alteration of gastric cancer cells towards CD44^+^/CD24^−^ cancer stem cell (CSC)-like cells. Quantification proteomics analysis revealed that the differentially expressed proteins in gastric cancer cells were mainly involved in the AKT/mTOR and MEK/ERK signalling pathways.

**Conclusions:**

SPARC expression in GCAFs is a useful prognostic factor in patients with gastric cancer. Low expression of GCAF-derived SPARC can lead to CSC transformation and 5-FU resistance. Additionally, the AKT/mTOR and MEK/ERK signalling pathways may participate in the malignant process.

**Electronic supplementary material:**

The online version of this article (10.1186/s12935-019-0844-8) contains supplementary material, which is available to authorized users.

## Background

Gastric cancer is one of the most prevalent fatal diseases worldwide, with a mortality rate behind that of only lung cancer and colorectal cancer according to data from Global Cancer Statistics 2018 [1]. The resistance of gastric cancer to chemotherapeutic drugs partially accounts for the failure of radical and palliative treatment, which leads to poor prognosis. The mechanisms underlying this phenomenon mainly include the efflux function of ATP-binding cassette membrane transporters, autophagy, and cancer stem cells (CSCs), which participate in many resistance processes [2, 3]. Therefore, identifying effective factors to regulate CSCs is a key to overcoming drug resistance.

Secreted protein acidic and rich in cysteine (SPARC), also called osteonectin, is a 32 kDa extracellular matrix glycoprotein that plays roles in tissue remodelling and cell–matrix crosstalk. Recently, emerging evidence has shown that SPARC could also affect the malignant process in various solid tumours [4, 5]. However, the functions of SPARC are highly heterogeneous among tumour types. Tumour cell-derived SPARC was reported to inhibit cell proliferation, invasion and angiogenesis in gastric cancer [6]. In addition, Chen et al. [7] found that SPARC was mainly expressed in gastric cancer-associated fibroblasts (GCAFs) and that the low expression level of SPARC in gastric cancer cells may be due to aberrant methylation of the SPARC gene promoter.

As the main source of SPARC, GCAFs also play an important role in the tumour microenvironment, and our previous work suggested that activated GCAFs contribute to the malignant phenotype and 5-fluorouracil (5-FU) resistance via paracrine action in gastric cancer [8]. However, most studies have concentrated on tumour cell-derived SPARC, and the role of GCAF-derived SPARC in gastric cancer progression is poorly understood. In this study, we aimed to investigate the correlations of GCAF-derived SPARC with clinicopathological features in gastric cancer, to explore the underlying mechanisms of GCAF-derived SPARC in regulating stemness transformation and 5-FU resistance in gastric cancer, and to demonstrate the prognostic and therapeutic value of SPARC.

## Materials and methods

### Clinical materials

The present study included 192 patients with primary gastric cancer. The histological types were confirmed by pathologists from the Department of Pathology, Peking University First Hospital. All patients underwent radical or palliative resection for gastric cancer, and 98 patients received regular follow-up for 5 years. We excluded patients who received neoadjuvant treatment before surgery. Paraffin-embedded tumour samples were obtained from the Peking University First Hospital Surgical Tumour Bank. In addition, fresh tumour samples used for primary culture were obtained from another three patients in 2017. The study protocol conformed to the ethical guidelines of the Peking University First Hospital Biomedical Research Ethics Committee (No. 2017-37). Informed consent agreements were obtained from all patients.

### Immunohistochemical detection of SPARC

Paraffin-embedded tumour tissues were cut into 4-µm-thick sections and fixed on slides. An anti-SPARC antibody (1:100 dilution, CST, MA, USA) was used to detect SPARC, and horseradish peroxidase (HRP)-conjugated goat anti-rabbit IgG (ZSGB-BIO, Beijing, China) was used as the secondary antibody. Sections incubated with phosphate-buffered saline (PBS) instead of the primary antibody were used as the negative controls. The DAB staining system was used to visualize SPARC expression. The status of SPARC expression was evaluated independently by three researchers, primarily by the staining intensity but also by the percentage of stained cells [9]. Briefly, the intensity was scored as follows: 0, no staining; 1, weak staining; 2, moderate staining; and 3, strong staining. The staining percentage was scored as follows: 0, complete absence of staining or staining in ≤ 5% of cells of the same type; 1, staining in 6% to 25% of cells; 2, staining in 26% to 50% of cells; and 3, staining in > 50% of cells. The sum of these two scores represented the expression level of SPARC: scores of < 4 were categorized as low expression; scores of ≥ 4, high expression. The levels of SPARC expression were calculated separately in cancer cells and cancer-associated fibroblasts.

### Primary culture of GCAFs

A modified method for the primary culture of GCAFs was developed in our previous study [8]. Briefly, tumour samples were dissected from cancer foci during surgery under aseptic conditions and processed within 1 h. To remove most pathogenic microbes, samples were washed in PBS containing 100 U/mL penicillin and 100 µg/mL streptomycin (Thermo Fisher Scientific, MA, USA). Moreover, Normocin, proven to effectively prevent mycoplasma, bacterial and fungal contamination, was added at a concentration of 200 µg/mL. Tissue fragments were cut into 1-mm^3^ pieces and digested with 0.25% trypsin for 30 min. The mixture of tissues and digestion liquid was transferred into flasks for subsequent culture. Cells were incubated in 5% CO_2_ at 37 °C. The concentration of foetal bovine serum (FBS, Thermo Fisher Scientific, MA, USA) was reduced to 5% after the second passage. Density gradient centrifugation was applied to purify GCAFs in stationary phase.

### Cell line

The human gastric cancer cell lines BGC-823 and SGC-7901 were obtained from the Cancer Institute of the Chinese Academy of Medical Sciences, and the MKN-45 cell line was obtained from the American Type Culture Collection (ATCC). The culture medium was composed of Dulbecco’s modified Eagle’s medium (DMEM, Thermo Fisher Scientific, MA, USA) or Roswell Park Memorial Institute 1640 (RPMI 1640, Thermo Fisher Scientific, MA, USA) medium supplemented with 10% FBS and antibiotics. Cells were cultured at 37 °C with 5% CO_2_ supplementation.

### Lentivirus infection

The plasmids pLenti-U6-EGFP-Puro-sh and pLenti-U6-EGFP-Puro-nc were purchased from Vigene Biosciences (Shandong, China). The sequence used for SPARC knockdown was 5′-GCAGAGGTGACTGAGGTATCT-3′, while the nontargeting sequence was 5′-GCACCCAGTCCGCCCTGAGCAAA-3′. The psPAX2-pMD2.G system was applied for lentiviral packaging according to the manufacturer’s instructions. Supernatant containing the virus was harvested 48 h after packaging. The supernatant was filtered through a 0.45 μm filter and incubated with PEG-8000 (Solarbio, Beijing, China) overnight. After centrifugation at 10,000 rpm for 30 min, the concentrated lentivirus was taken as the precipitate resuspended in DMEM. The dose of lentivirus added to GCAFs was determined by the optimal multiplicity of infection (MOI) value, and cells were cultured at 37 °C with 5% CO_2_, followed by replacement of the medium with fresh medium. Green fluorescence was typically visualized after 2–4 days depending on the growth conditions of the GCAFs. The established GCAFs with SPARC knockdown and the negative control GCAFs are denoted GCAF-sh and GCAF-nc, respectively. In addition, the mScarlet-labelled BGC-823 and SGC-7901 cell lines were established by this approach.

### Western blot analysis

The expression levels of proteins in GCAFs and cancer cells were assessed as follows. Total cellular protein samples were prepared from cell lysates in lysis buffer. After the protein concentration of each sample was adjusted, sodium dodecyl sulfate (SDS)–polyacrylamide gel electrophoresis was carried out to separate proteins. Subsequently, the protein bands obtained were transferred to polyvinylidene difluoride (PVDF) membranes. Membranes were incubated with specific primary antibodies against SPARC (1:1000 dilution, CST, MA, USA), PARP (1:1000 dilution, CST, MA, USA), caspase 3 (1:1000 dilution, CST, MA, USA), Bak (1:1000 dilution, CST, MA, USA), Bax (1:1000 dilution, CST, MA, USA), tubulin (1:1000, CST, MA, USA), and GAPDH (1:1000, CST, MA, USA) at 4 °C overnight. GAPDH and tubulin were used as the internal controls. The expression levels of the target proteins were assessed via an ECL detection system (Merck, Darmstadt, Germany) on a Syngene GeneGenius gel imaging system (Syngene, Cambridge, UK). And the relative changes of protein expression were analysed using Image J software.

### Flow cytometric analysis

An Annexin V-FITC/PI Apoptosis Assay Kit (KeyGen, Jiangsu, China) was employed to detect apoptotic cells. Gastric cancer cells were co-cultured for 72 h with conditioned medium (CM) from GCAFs (7-day culture) in the presence of 5-FU (1 μg/mL). After incubation with annexin V-FITC and propidium iodide (PI) for 5 min, the apoptotic status of cells was analysed using flow cytometry. For phenotypic analysis of CSCs, cancer cells co-cultured with CM were dispersed into a single-cell suspension and blocked with Fc Receptor Blocking Solution (Biolegend, CA, USA) for 10 min. Antibodies against human CD24 (FITC, Biolegend, CA, USA) and CD44 (PE, Biolegend, CA, USA) were added at dilutions of 1:10 and 1:100, respectively, and incubated at room temperature in the dark. Phenotypic analysis was performed on a FACSCalibur flow cytometer (BD Biosciences, NJ, USA). Each intervention was performed with three replicates.

### Three-dimensional (3D) cell co-culture for tumour sphere formation

To mimic the in vivo spatial structure, Perfecta3D hanging drop plates (Sigma, MO, USA) were used for 3D co-culture of gastric cancer cells and GCAFs. In brief, equal numbers of mScarlet-labelled SGC-7901/BGC-823 cells and GCAFs were mixed and diluted to a concentration of 2.5 × 10^4^ cells/mL. Then, 40 μL of the cell suspension was added to each hanging drop plate well and cultured for 72 h. An additional 10 μL of fresh medium was added after 48 h. Cells in the drug response group were treated with 5-FU (1 μg/mL) for 48 h. The cell suspension was transferred and harvested in the receiving plates. The spheroid colonies containing mScarlet-labelled gastric cancer cells and GFP-labelled GCAFs were confirmed and imaged by confocal laser scanning microscopy.

### Label-free quantification proteomics

MKN-45 cells were cultured in CM from GCAF-nc and GCAF-sh cells for 72 h before label-free quantification proteomics analysis. Briefly, protein (200 μg in each sample) from GCAFs was extracted from tissue samples using SDT lysis buffer and quantified with a BCA Protein Assay Kit (Bio-Rad, USA). After digestion with the Filter-Aided Sample Prep method described by Wisniewski et al. [10], liquid chromatography-tandem mass spectrometry (LC–MS/MS) peptide analysis was performed on a Q Exactive Plus mass spectrometer coupled to an Easy 1200 nLC liquid chromatography system (Thermo Fisher Scientific, MA, USA). Next, the MS data were analysed using MaxQuant software version 1.6.0.16 and were searched against the UniProt database for *Homo sapiens* (169,753 total entries, downloaded 18/2/2019). Analyses of the bioinformatics data were carried out with Perseus, Microsoft Excel and R statistical computing software. For sequence annotation, information was extracted from the UniProtKB/Swiss-Prot database, the Kyoto Encyclopedia of Genes and Genomes (KEGG), and the Gene Ontology (GO) resource. Construction of protein–protein interaction (PPI) networks was also conducted by using the online STRING database.

### Statistical analysis

Correlations between SPARC expression and clinicopathological features were evaluated using the χ^2^ or Kruskal–Wallis test, if appropriate. Kaplan–Meier analysis was applied to calculate the survival duration, and the significance between groups was analysed using the log-rank test. Cox regression analysis was employed to compute multivariate hazard ratios (HRs) for the parameters. A t-test was used to compare the results from the two groups. *P *< 0.05 was considered significant, and the results of all tests noted above were analysed using SPSS 24.0 software. The quantitative protein ratios were weighted and normalized by the median ratio in MaxQuant software. Only proteins with a fold change of ≥ 2.0 and a *P* value of < 0.05 were considered significantly differentially expressed. GO and KEGG enrichment analyses were conducted with Fisher’s exact test, and false discovery rate correction for multiple hypothesis testing was performed.

## Results

### Relationship between clinicopathological features and SPARC expression in gastric cancer

**Fig. 1:**
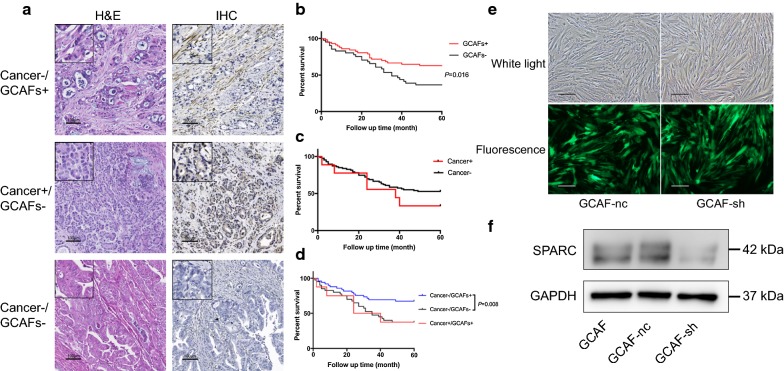
Correlations between OS and SPARC expression and the establishment of the GCAF strain with SPARC knockdown. **a** Immunohistochemical staining of SPARC and H&E staining of gastric cancer tissues (scale bar = 100 μm). **b** The Kaplan–Meier OS curve with respect to GCAF-derived SPARC levels. **c** The Kaplan–Meier OS curve with respect to cancer cell-derived SPARC levels. **d** The Kaplan–Meier OS curve with respect to the combined expression of SPARC in gastric cancer. **e** The morphology of GCAF-nc and GCAF-sh cells as observed via bright field and fluorescence microscopy (scale bar = 1 mm). **f** The western blotting results showed significant inhibition of SPARC expression in GCAF-sh cells compared with that in GCAFs and GCAF-nc cells

A total of 192 patients were enrolled in this study and their tumour tissues were evaluated using immunohistochemistry (IHC). Haematoxylin and eosin (H&E) staining was performed to distinguish the various cellular components. SPARC was mainly expressed in GCAFs (47.4%) and was rarely expressed in cancer cells (11.5%, Fig. 1a). Furthermore, we investigated the correlations between clinicopathological features and both GCAF-derived SPARC and cancer cell-derived SPARC (Table 1). GCAF-derived SPARC was significantly associated with tumour differentiation, and low expression indicated poor differentiation in gastric cancer (*P *= 0.003). However, cancer cell-derived SPARC was not significantly associated with histological type, grade, tumour location, cancer embolus, or clinical stage.

**Table 1:** Correlations between SPARC staining and clinicopathological features of patients with gastric cancer

Parameter	No.	Stromal SPARC expression		*P* value	Cancer SPARC expression		*P* value
		High	Low		High	Low	
Gender							
Female	36	14 (39%)	22 (61%)	0.257	3 (8%)	33(92%)	0.717
Male	156	77 (49%)	79 (51%)		19 (12%)	137 (88%)	
Age							
< 70	142	64 (45%)	78 (55%)	0.277	18 (13%)	124 (87%)	0.372
≥ 70	50	27 (54%)	23 (46%)		4 (8%)	46 (92%)	
Histological type							
Adenocarcinoma	153	67 (44%)	86 (56%)	0.048	16 (10%)	137 (90%)	0.561
Other type	39	24 (62%)	15 (38%)		6 (15%)	33 (85%)	
Grade							
Well differentiated	18	12 (67%)	6 (33%)	0.003	5 (28%)	13 (72%)	0.074
Moderately differentiated	67	37 (55%)	30 (45%)		6 (9%)	61 (91%)	
Poorly differentiated	94	34 (36%)	60 (64%)		7 (7%)	87 (93%)	
Tumor location							
Fundus-cardia	26	17 (65%)	9 (35%)	0.195	1 (4%)	25 (96%)	0.375
Body	28	21 (75%)	7 (25%)		8 (29%)	20 (71%)	
Antrum	54	31 (57%)	23 (43%)		5 (9%)	49 (91%)	
Diffused	12	5 (42%)	7 (58%)		0 (0)	12 (100%)	
Degree of invasion							
T1	5	3 (60%)	2 (40%)	0.907	2 (40%)	3 (60%)	0.825
T2	25	16(64%)	9 (36%)		2 (8%)	23 (92%)	
T3	59	35 (59%)	24 (41%)		6 (10%)	53 (90%)	
T4	31	20 (65%)	11 (35%)		4 (13%)	27 (87%)	
Lymph node metastasis							
0	27	20 (74%)	7 (26%)	0.277	6 (22%)	21 (78%)	0.237
1–2	18	9 (50%)	9 (50%)		1 (6%)	17 (94%)	
3–6	34	22 (65%)	12 (35%)		3 (9%)	31 (91%)	
7 or more	41	23 (56%)	18 (44%)		4 (10%)	37 (90%)	
Cancer embolus							
Yes	50	30 (60%)	20 (40%)	0.751	4 (8%)	46 (92%)	0.290
No	70	44 (63%)	26 (37%)		10 (14%)	60 (86%)	
Stage							
I	41	17 (41%)	24 (59%)	0.063	6 (15%)	35 (85%)	0.248
II	77	32 (42%)	45 (58%)		10 (13%)	67 (87%)	
III	74	42 (57%)	32 (43%)		6 (8%)	68 (92%)	

### Overall survival (OS)

Figure 1b–d show the Kaplan–Meier survival curves for 98 patients with gastric cancer. Low expression of GCAF-derived SPARC indicated reduced OS compared with that in the high expression group (χ^2^ = 5.819, *P *= 0.016). However, cancer cell-derived SPARC was not statistically correlated with OS based on the log-rank test (χ^2^ = 1.055, *P *= 0.304). Combined analysis of SPARC from heterogeneous sources revealed that the Cancer^−^/GCAF^+^ group had the best prognosis among the groups; the difference in OS between this group and the Cancer^−^/GCAFs^−^ group was statistically significant (χ^2^ = 7.071, *P *= 0.008). Univariate analysis showed that OS was significantly correlated with GCAF-derived SPARC expression, tumour invasion, lymph node metastasis, and cancer embolus. Multivariable Cox regression analysis demonstrated that GCAF-derived SPARC was an independent predictive factor for OS in gastric cancer (HR = 0.418, *P *= 0.015, Table 2).

**Table 2:** Univariate and multivariate Cox multiple regression analysis of overall survival after surgery in patients with gastric cancer

Parameter	Univariate			Multivariate		
	HR	95% CI	*P* value	HR	95% CI	*P* value
SPARC in GCAFs						
Positive	0.502	0.279–0.904	0.016	0.418	0.207–0.841	0.015
SPARC in cancer cells						
Positive	1.556	0.561–4.315	0.304	2.489	0.671–9.238	0.173
Histological type						
Adenocarcinoma	0.755	0.4043–1.409	0.349	0.744	0.353–1.570	0.438
Grade						
Poorly differentiated	1.512	0.815–2.804	0.180	1.358	0.710–2.597	0.355
Degree of invasion						
Deep	2.361	1.259–4.426	0.030	1.714	0.726–4.043	0.219
Lymph node metastasis						
Positive	3.708	1.927–7.136	0.007	1.640	0.523–5.140	0.396
Cancer embolus						
Positive	1.847	1.025–3.329	0.032	1.543	0.798–2.982	0.197

### Establishment of GCAF strains with SPARC knockdown

GCAFs obtained from gastric cancer patients were successfully cultured via the modified primary culture method. To elucidate the role of SPARC in GCAFs, a lentivirus infection method was used to establish GCAF cells with stable SPARC knockdown, which were named GCAF-sh cells. GCAF cells transfected with a nontargeting sequence, named GCAF-nc cells, were used as the negative control. The green fluorescence indicated a high rate of infection, and the western blot showed significant inhibition of SPARC expression in the GCAF-sh strain (Fig. 1e, f).

### Effect of GCAF-derived SPARC on 3D gastric tumour growth and 5-FU response in vitro

**Fig. 2:**
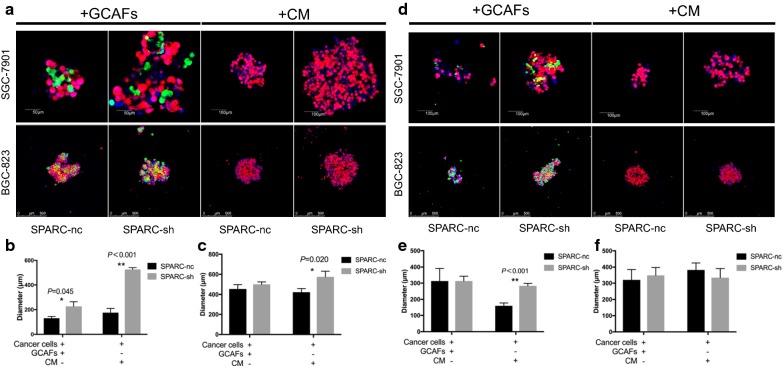
Effect of GCAF-derived SPARC on 3D gastric tumour growth and the 5-FU response in vitro. Tumour growth: **a** Confocal laser scanning microscopy of 3D gastric cancer tumour spheres (the scale bar was adjusted according to the actual sphere sizes). **b** Comparison of the sphere diameters in SGC-7901 cells. **c** Comparison of the sphere diameters in BGC-823 cells. 5-FU response: **d** Confocal laser scanning microscopy of 3D gastric cancer tumour spheres (the scale bar was adjusted according to the actual sphere sizes). **e** Comparison of the sphere diameters in SGC-7901 cells. **f** Comparison of the sphere diameters in BGC-823 cells. *P < 0.05, **P < 0.01

To mimic the 3D growth pattern of gastric cancer in vivo, Perfecta3D hanging drop culture techniques were applied to establish the co-culture model, and the 3D-reconstruction sphere movies were attached in Additional files 1 and 2. Gastric cancer cells were co-cultured with GCAFs or cultured in CM from GCAFs for 72 h, and images were acquired via fluorescence microscopy (Fig. 2a–c). The average diameter of SGC-7901 spheres co-cultured with GCAF-sh cells was significantly larger than that of SGC-7901 spheres co-cultured with GCAF-nc cells (226.7 ± 21.86 μm vs 130.0 ± 10.00 μm, *P *= 0.045). In addition, the average diameter of SGC-7901 spheres cultured in CM from GCAF-sh cells was larger than that of SGC-7901 spheres cultured in CM from control cells (526.7 ± 8.82 μm vs 175.0 ± 25.00 μm, *P *< 0.001). The same trend was observed for BGC-823 spheres. 5-FU was introduced into the 3D co-culture model to explore the effect of GCAF-derived SPARC on 5-FU resistance in gastric cancer (Fig. 2d–f). After culture with 5-FU for 72 h, the average diameter of SGC-7901 spheres cultured in CM from GCAF-sh cells was significantly larger than that of SGC-7901 spheres cultured in CM from GCAF-nc cells (283.3 ± 8.82 μm vs 160 ± 10.00 μm, *P *< 0.001).

### Effect of GCAF-derived SPARC on 5-FU-induced apoptosis in gastric cancer

**Fig. 3:**
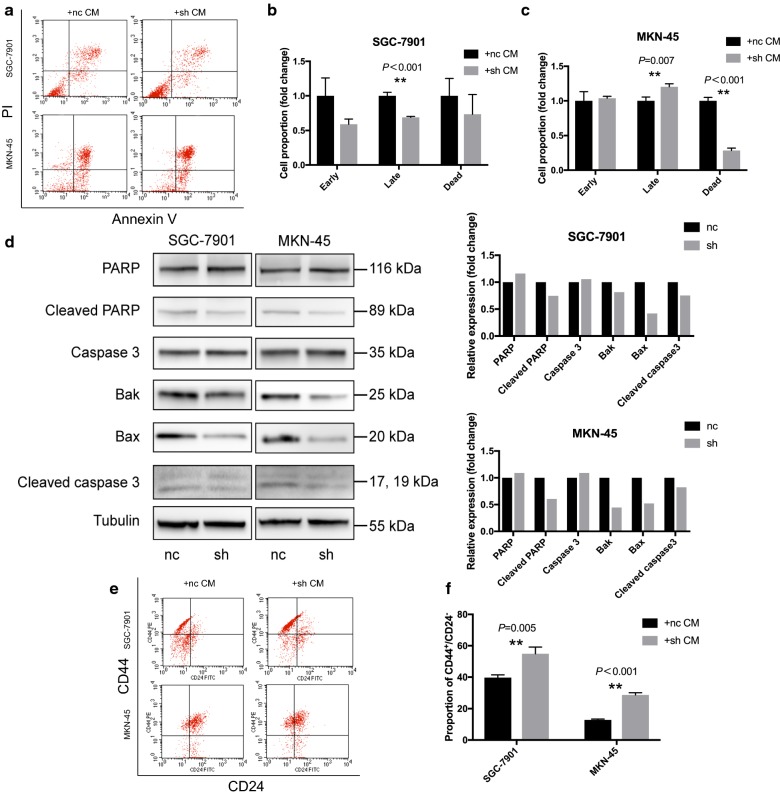
Effect of GCAF-derived SPARC on 5-FU-induced apoptosis and stemness transformation in gastric cancer. **a** Flow cytometric apoptosis analysis. **b** Comparison of the proportion of apoptotic SGC-7901 cells after treatment with CM from GCAFs. **c** Comparison of the proportion of apoptotic MKN-45 cells after treatment with CM from GCAFs. **d** Expression levels of mitochondrial pathway apoptosis-associated proteins in gastric cancer cells after treatment with 5-FU for 72 h. **e** Flow cytometric analysis of stemness biomarkers. **f** Comparison of the CD44^+^/CD24^−^ cell population in GCAF-sh cells and the GCAF-nc cells. **P < 0.01

Flow cytometry and western blotting were applied to investigate alterations in apoptosis after treatment with 5-FU in gastric cancer cells cultured in CM from GCAFs. A decrease in the proportion of late apoptotic SGC-7901 cells was observed in the GCAF-sh treatment group compared with that in the GCAF-nc treatment group (21.73 ± 0.24% vs 31.60 ± 0.92%, *P *< 0.001, Fig. 3a, b). However, although the proportion of late apoptotic MKN-45 cells increased in the GCAF-sh treatment group, the proportion of dead cells was significantly reduced compared with that in the GCAF-nc treatment group (2.10 ± 0.15% vs 7.36 ± 0.21%, *P *< 0.001), indicating that the 5-FU response was shifted from cell death back to apoptosis (Fig. 3a, c). Western blot analysis showed that after treatment with 5-FU, the expression of Bax, Bak, cleaved caspase 3, and cleaved PARP was downregulated in the GCAF-sh treatment groups of gastric cancer cells (Fig. 3d).

### GCAF-derived SPARC decreased the CD44^+^/CD24^−^ cell population of gastric cancer

**Fig. 4:**
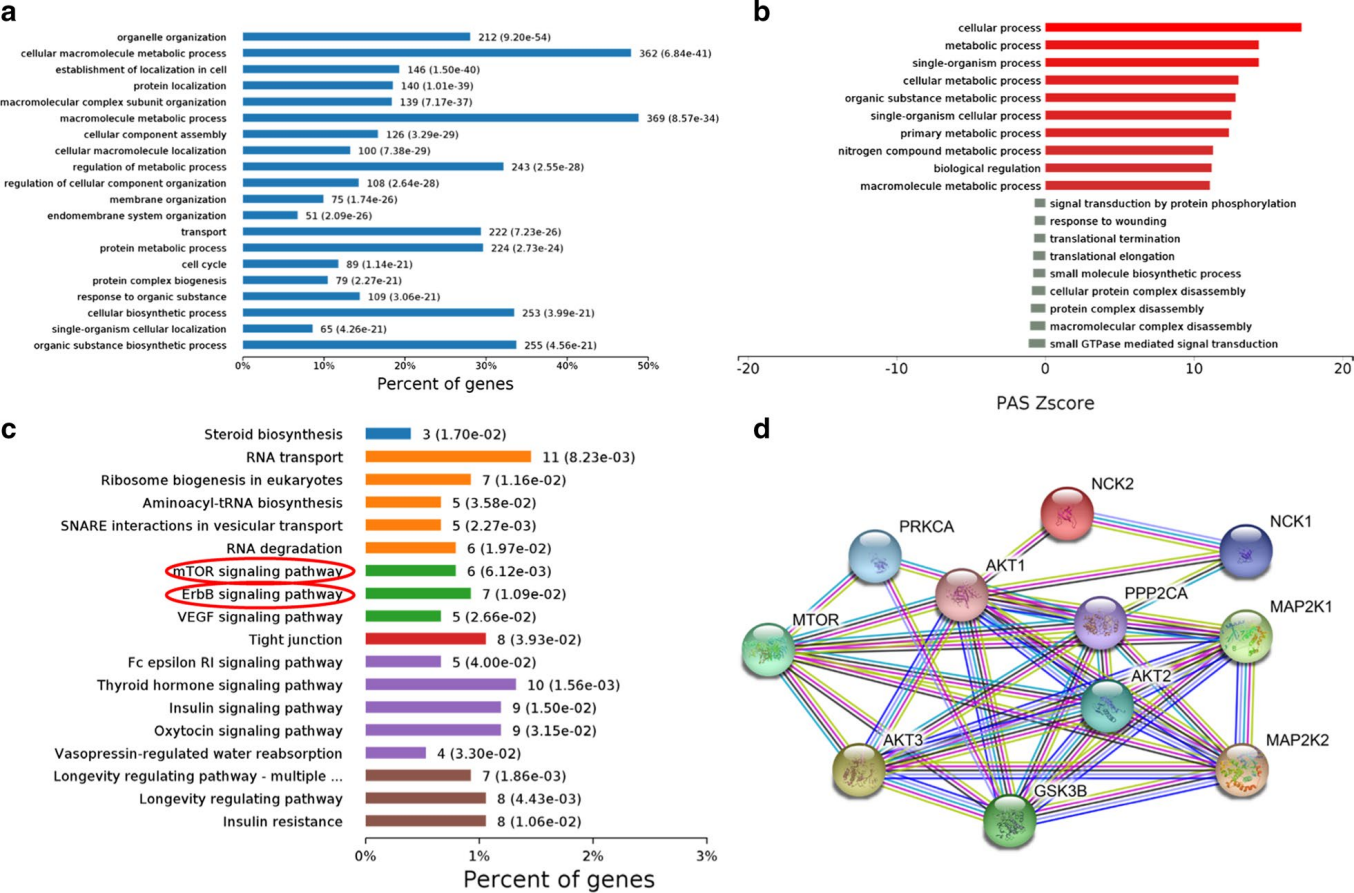
Quantification proteomics analysis. **a** GO enrichment analysis. **b** Regulatory status of differential biological processes. The terms in red were enriched in upregulated proteins, and the terms in green were enriched in downregulated proteins. **c** KEGG differential pathway enrichment analysis. **d** PPI analysis of DAPS involved in the ErbB-MEK/ERK pathway and the AKT/mTOR pathway

The gastric cancer cell lines MKN-45 and SGC-7901 were cultured in CM from GCAFs for 72 h, and flow cytometry was performed to evaluate the percentage of CSC-like cells based on the expression of the cell surface markers CD44 and CD24 (Fig. 4e, f). CD44^+^/CD24^−^ cells were considered the CSC population [11, 12]. After treatment with CM from GCAF-sh cells, the percentage of CD44^+^/CD24^−^ cells among the total population of SGC-7901 cells was significantly higher than that among SGC-7901 cells treated with CM from GCAF-nc cells (54.90% vs 39.72%, *P *= 0.005). The same trend was observed for MKN-45 cells (28.78% vs 12.85%, *P *< 0.001). Decreasing the expression of SPARC in GCAFs exerted a favourable effect in the phenotypic alteration of gastric cancer cells towards CSC-like cells.

### Analysis of differentially abundant protein species (DAPS) in gastric cancer cells after treatment with CM from GCAFs

To investigate the changes in the expression levels of gastric cancer-associated proteins after the inhibition of GCAF-derived SPARC, MKN-45 cells treated with CM from GCAF-sh and GCAF-nc cells were analysed via label-free quantification proteomics. Based on the threshold for screening DAPS, 192 differential proteins were identified and quantified, among which 125 were upregulated and 67 were downregulated. GO analysis indicated that the DAPS were mainly enriched in biological process and cell component terms (Fig. 4a). Additionally, many metabolic processes were upregulated in MKN-45 cells after treatment with GCAF-sh CM (Fig. 4b). To further identify the significant differences in cancer-associated pathways between the two groups, KEGG analysis was performed on the DAPS (Fig. 4c). We identified two differentially enriched pathways: the AKT/mTOR signalling pathway (*P *= 0.006) and the ErbB-MEK/ERK pathway (*P *= 0.011). The DAPS involved in these pathways mainly included AKT, MTOR, PRKCA, PPP2CA, GSK3B, p70S6K, MEK, ERK, and NCK. PPI analysis showed a tight and network-like connection among these DAPS (Fig. 4d).

## Discussion

The tumour microenvironment, which includes cellular components and a non-cellular matrix, is an interactive system contributing to carcinogenesis and cancer development. As one of the most important kinds of stromal cells, CAFs have been reported not only to exert effects on cancer cells but also to interact with other cells within the microenvironment, such as tumour-associated macrophages and granulocytes [13]. CAFs can regulate malignant behaviours via direct contact with cancer cells or via paracrine action to secrete chemokines and exosomes as interference mechanisms [14]. SPARC is an extracellular matrix glycoprotein and acts as a bridge between the parenchyma and mesenchyma. Overexpression of SPARC disrupts the recognition of VEGF by its receptor, inhibits the activation of ERK1/2, and consequently limits the proliferation of cancer cells [15]. Additionally, the SPARC promoter can drive the expression of a suicidal gene that can suppress tumour cell growth [16]. Our previous work revealed that SPARC was mainly expressed in GCAFs in gastric cancer. However, most evidence supporting the role of SPARC in solid tumours is based on cancer cell-derived SPARC [17–19]. SPARC derived from different cellular origins is reported to be highly heterogeneous, and SPARC expressed by different cells may even lead to opposite clinical outcomes [20]. The confirmed effects and underlying mechanisms of GCAF-derived SPARC in gastric cancer remain to be demonstrated.

In the present study, to reveal the clinical significance of GCAF-derived SPARC, we used immunohistochemical staining to evaluate the expression level of SPARC in 192 gastric carcinoma samples. SPARC was mainly expressed in GCAFs and was rarely expressed in cancer cells. Low expression of GCAF-derived SPARC indicated poor differentiation in gastric cancer. Furthermore, high expression of GCAF-derived SPARC was associated with improved 5-year OS and was an independent predictive factor. However, in the group with cancer cell-derived SPARC expression, no significant differences were observed among the clinicopathological parameters. A meta-analysis including 10 studies that focused on SPARC expression in gastric cancer showed that SPARC overexpression was highly correlated with reduced OS [21]. However, this analysis was based on overall expression in tumour foci, and different detection methods were evaluated together, possibly explaining the high heterogeneity of the pooled effect sizes. Our results indicate that the cellular origin of SPARC should be considered when analysing clinical significance.

Drug resistance is a powerful barrier to postoperative adjuvant treatment and affects the long-term survival of gastric cancer patients. CAFs could contribute to this phenomenon in multiple ways, according to recent studies in pancreatic cancer. For example, drug scavenging by fibroblasts increases intratumoural gemcitabine accumulation in cancer, making the drug unavailable to murine pancreatic cancer cells [22]. Additionally, CAFs can enhance the functions of CSCs through the production of type I collagen to activate integrin-FAK signalling in pancreatic cancer [23]. Our 3D co-culture model was modified to facilitate the observation of stemness phenotypes, spherical growth and 5-FU resistance in gastric cancer. After co-culture with GCAFs or culture in CM from GCAFs, tumour spheres were larger in the GCAF-sh group than in the GCAF-nc group. Moreover, the 5-FU resistance assays demonstrated an increasing trend in SGC-7901 cells cultured with CM from GCAF-sh cells. Subsequent apoptosis analysis revealed that SPARC inhibition in GCAFs suppressed late apoptosis and cell death processes in gastric cancer cells. Additionally, the expression of apoptosis-related proteins was downregulated in cancer cells after treatment with CM from GCAF-sh cells, indicating an anti-mitochondrial pathway apoptosis effect. Our data showed that knocking down SPARC in GCAFs promoted 5-FU resistance in gastric cancer cells.

Surface biomarkers are the most common tools for the identification and isolation of CSCs from other cells. CD44 is a membrane glycoprotein that induces signal transduction among cells, and its expression is correlated with migration, metastasis, and poor prognosis in several tumours [24, 25]. In gastric cancer, CD44 is considered a CSC biomarker for chemoresistance and progression [26]. CD24, another cell surface protein, is also involved in malignancy, but its prognostic value and clinical significance remain controversial [27]. CD44^+^/CD24^−^ breast cancer cells are widely accepted to act as the stem cell-like population. Xu et al. [28] found that CD44^+^/CD24^−^ gastric cancer cells also exhibit CSC features, such as self-renewal ability, tumourigenicity and 5-FU resistance. In the present study, after treatment with 5-FU in the presence of GCAFs, the percentage of CD44^+^/CD24^−^ gastric cancer cells was significantly elevated in the GCAF-sh group. Inhibition of SPARC expression in GCAFs facilitated the phenotypic alteration of gastric cancer cells towards CSC-like cells.

To further explore the molecular mechanisms of GCAF-derived SPARC in regulating stemness and 5-FU resistance, label-free quantification proteomics analysis was performed on gastric cancer cells treated with CM from GCAFs. Several DAPS potentially mediating relevant behaviours were identified. Bioinformatics analysis revealed that these DAPS were mainly involved in the AKT/mTOR and MEK/ERK signalling pathways, which are related to drug resistance and stemness. The serine/threonine kinase AKT transfers survival signals through phosphorylated mTOR, p70S6K, and 4EBP1 to inhibit the apoptosis of cancer cells, and p70S6K can also accelerate cell cycle progression in cancer cells [29]. Activation of the AKT/mTOR pathway leads to therapeutic resistance and is a poor prognostic factor in many tumours, which mainly relies on the binding of ligands to receptors in the cell membrane [30]. Many materials such as growth factors, cytokines, and hormones can serve as ligands for the activation of AKT. In the present study, the conditioned medium from GCAFs with SPARC knockdown could arouse AKT expression, and lead to the increasing abilities of stemness and 5-FU resistance in gastric cancer cell lines. We hypothesize that SPARC participates the regulations of paracrine actions in GCAFs, and the alterations of paracrine factors in CM cause the activation of AKT/mTOR in gastric cancer towards aberrant proliferation and anti-apoptosis.

**Fig. 5:**
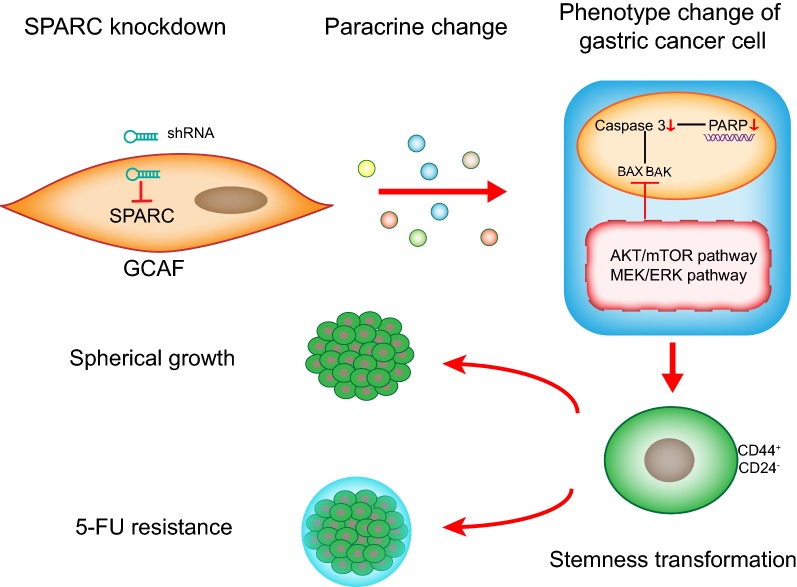
Graphical summary. SPARC knockdown in GCAFs can lead to CSC transformation and 5-FU resistance. The AKT/mTOR, MEK/ERK and other important signalling pathways may participate in this process

In addition, MEK/ERK (MAPK) signalling pathway is also correlated with the chemotherapeutic response. ERK, as a member of the MAPK family, regulates the cell growth and differentiation, and also contributes to malignant transformation when aberrantly activated. ERKs are phosphorylated when MEK is activated, and dimerized to translocate into the nucleus, leading to a series of phosphorylation and activation of transcription factors. Defects in the replication stress response are reported to stimulate the transformation of non-malignant cells into a CSC-like state, which is induced by signal transduction in the MEK/ERK pathway [31]. Additionally, targeted drugs such as selumetinib (AZD6244), an inhibitor of MEK, have the potential to suppress tumour development and resensitize cells to drug therapy [32]. Taken together, GCAF-derived SPARC might regulate stemness and the 5-FU response via these two pathways, and the underlying mechanisms need further investigation and verification (Fig. 5). The combination of GCAF-derived SPARC and pathway inhibitors may be a promising approach to reconsider in the adjuvant treatment of gastric cancer.

## Conclusions

In conclusion, SPARC expression in GCAFs is a useful prognostic factor in patients with gastric cancer. Low expression of GCAF-derived SPARC can lead to CSC transformation and 5-FU resistance. Additionally, the AKT/mTOR and MEK/ERK signalling pathways may participate in the malignant process.

## Additional files

**Additional file 1.** 3D-reconstruction movie of tumour sphere.

**Additional file 2.** 3D-reconstruction movie of tumour-GCAF symbiotic sphere.

## Data Availability

All data and materials are fully available without restriction. Data generated or analysed during this study are included in this published article.

## Abbreviations

ATCC: American Type Culture Collection
CAF: cancer-associated fibroblast
CM: conditioned medium
CSC: cancer stem cell
DAPS: differentially abundant protein species
DMEM: Dulbecco’s modified Eagle’s medium
FBS: foetal bovine serum
GCAF: gastric cancer-associated fibroblast
GO: Gene Ontology
H&E: haematoxylin and eosin
HR: hazard ratio
IHC: immunohistochemistry
KEGG: Encyclopedia of Genes and Genomes
LC–MS/MS: liquid chromatography–tandem mass spectrometry
MOI: multiplicity of infection
OS: overall survival
PBS: phosphate-buffered saline
PI: propidium iodide
PVDF: polyvinylidene difluoride
PPI: protein–protein interaction
RPMI: Roswell Park Memorial Institute
SDS: sodium dodecyl sulfate
SPARC: secreted protein acidic and rich in cysteine
3D: three-dimensional
5-FU: 5-fluorouracil
